# Three-year follow-up of physical activity in Norwegian youth from two ethnic groups: associations with socio-demographic factors

**DOI:** 10.1186/1471-2458-8-419

**Published:** 2008-12-22

**Authors:** Åse Sagatun, Elin Kolle, Sigmund A Anderssen, Magne Thoresen, Anne Johanne Søgaard

**Affiliations:** 1 Center for Child and Adolescent Mental Health, Eastern and Southern Norway, P.O. Box 4623 Nydalen, 0405 Oslo, Norway; 2 Norwegian School of Sport Sciences, Department of Sports Medicine, P.O. Box 4014, Ullevål Stadion, 0806 Oslo, Norway; 3 Institute of Basic Medical Sciences, Department of Biostatistics, University of Oslo, P.O. Box 1122 Blindern, N-0317 Oslo, Norway; 4 Instiute of General Practice and Community Medicine, Section for Preventive Medicine and Epidemiology, University of Oslo, P.O. Box 1130 Blindern, N-0318 Oslo, Norway; 5 Division of Epidemiology, Department of Chronic Diseases, Norwegian Institute of Public Health, P.O. Box 4404, Nydalen, 0403 Oslo, Norway

## Abstract

**Background:**

More research on factors associated with physical activity and the decline in participation during adolescence is needed. In this paper, we investigate the levels, change, and stability of physical activity during the late teens among ethnic Norwegians and ethnic minorities, and we examine the associations between physical activity and socio-demographic factors.

**Methods:**

The baseline (T1) of this longitudinal study included 10^th ^graders who participated in the youth part of the Oslo Health Study, which was carried out in schools in 2000–2001. The follow-up (T2) in 2003–2004 was conducted partly at school and partly by mail. A total of 2489 (1112 boys and 1377 girls) participated both at baseline and at follow-up. Physical activity level was measured by a question on weekly hours of physical activity outside of school. Socio-demographic variables were collected by questionnaire and from data obtained from Statistics Norway. Analysis of variance was used to study the level of and changes (T1 to T2) in physical activity, and the associations between physical activity and socio-demographic factors. Stability in physical activity was defined as the percentage of students reporting the same physical activity both times.

**Results:**

Boys were more active than girls at age 15 and 18 years, independent of ethnic background. Among girls, ethnic Norwegians were more active than ethnic minorities. Hours per week spent on physical activity declined in all groups during the follow-up period. Few associations were found between physical activity and socio-demographic factors in both cross-sectional and longitudinal data. Among the ethnic minority girls, 65% reported being physically active 0–2 hours per week at baseline, and 82% of these girls reported the same level at follow up.

**Conclusion:**

The association between physical activity and ethnicity at age 15 years remained the same during the follow-up. Few associations were found between physical activity and socio-demographic variables. A large proportion of ethnic minority girls reported a persistently low physical activity level, and this low participation rate may need special attention.

## Background

Regular physical activity is important for healthy growth and development of children and adolescents. It helps build and maintain healthy bones, muscles, and joints, and enhances psychological well-being [1]. Regular physical activity contributes to the control of body weight, which is important because the prevalence of obesity is increasing [2]. Mid-adolescence is an important period in young people's life as adult patterns of health behaviours start to emerge. Habits of, and attitudes towards, physical activity developed during childhood are assumed to continue through adolescence and into adulthood [3,4]. Despite the importance of physical activity in youth, studies show consistently that participation in physical activity declines during adolescence. This decline has been reported in both cross-sectional [5-7] and longitudinal studies [8-17], and in studies using both self-reported measures [5,8-13] and objective measures of physical activity [6,7,17]. Knowledge about the factors related to this decline in physical activity is scarce.

With an increasing influx of immigrants to the Western parts of the world, cultural and ethnic background has become an important variable when studying health and health behaviour from the public health perspective. Few studies have investigated the relationship between physical activity and ethnicity in adolescence. Most studies have reported that ethnic minorities are less active than their majority counterparts [13,18-20], whereas others have not found any association [21] or have found that ethnic minorities are more physically active [16].

In addition to ethnicity, socio-economic status (SES) is an important factor regarding physical activity [22]. In children and adolescents, the association between SES and physical activity is inconsistent [23-26]. The controversy relates to whether SES and physical activity level are positively associated [20,21,24-27] or not related at all [24-26,28]. The differences between studies may reflect differences in the methods used to measure SES and physical activity and the subgroup studied [24-26].

Few longitudinal population-based studies in adolescents have focused on the relationship between physical activity and ethnicity while also considering socio-demographic factors [20]. In a longitudinal population-based study of about 2500 adolescents for 3 years, of which 20% had an ethnic minority background, we collected data about physical activity, ethnicity, and socio-demographic factors. The aims of this study were: (1) to estimate and compare levels of physical activity in ethnic Norwegian and ethnic minority youth at ages 15 and 18 years; (2) to examine the association between socio-demographic factors and physical activity in the two groups at 15 and 18 years; (3) to describe changes in, and stability of, physical activity in ethnic Norwegian and ethnic minority youth after three years of follow-up; and (4) to examine the relationships between socio-demographic factors and change in physical activity over the three years in the two ethnic groups.

## Methods

### Baseline study (T1)

All students in grade 10 (aged 15–16 years, later referred to as 15 years) in Oslo during the school years 1999–2000 and 2000–2001 were invited to enter the youth part of the Oslo Health Study. This was a questionnaire study conducted in schools. All parents received written information, and the students completed a consent form before participation. The students completed questionnaires during two school classes. For those not present on the day of the survey, questionnaires were left for them at school. A copy was mailed to the home address of those who did not respond, together with a stamped return envelope. A more detailed description has been published elsewhere [29,30]. From the total population of 10^th ^graders (both 1999–2000 and 2000–2001), 7343 (88%) participated. The 3811 subjects participating in the year 2000–2001 comprised the baseline of our longitudinal study. During the school year 2003–2004, a second study was conducted with the same adolescents.

### Follow-up study (T2)

The follow-up study was carried out partly as a school-based survey and partly through mail, as described elsewhere [30,31]. The procedure for the school-based part of the study was similar to the baseline procedure. All 32 secondary high schools in Oslo took part, and the senior year students (aged 18–19 years, later referred to as 18 years) completed the questionnaire during one school class. The participants in the baseline study (year 2000–2001) who were not enrolled in the senior year of secondary high school in Oslo and who had consented to participate in the follow-up were invited to participate by mail.

### Study population

**Figure 1:**
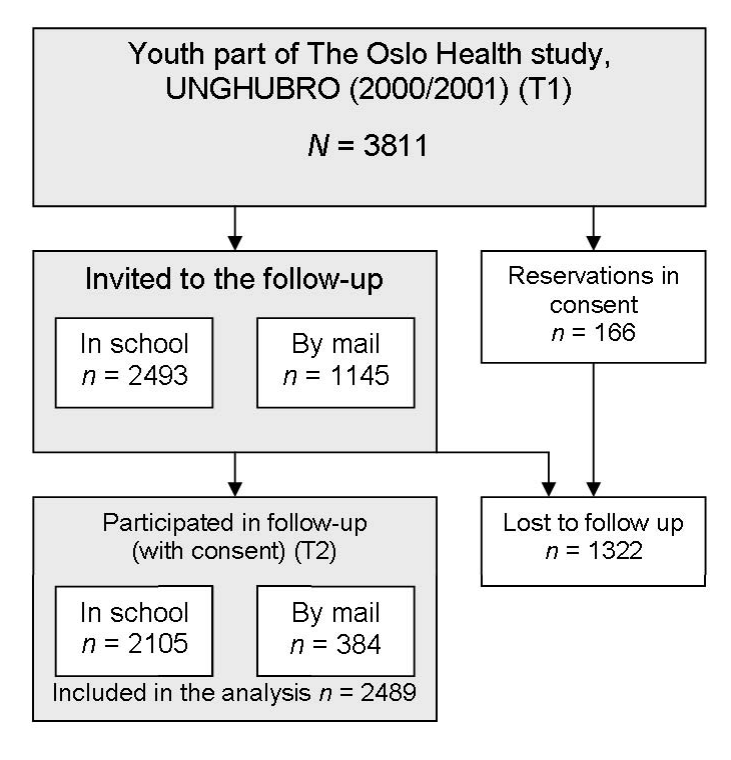
**Flow sheet of the study population**.

Of the 3811 participants in the baseline study, 2489 (65.3%) participated in the follow-up and gave their consent to link their data between the two surveys. Only the adolescents who participated in both surveys were included in our analyses (1,112 boys and 1,377 girls) (Figure 1). Of the participants in the follow-up, 20% had an ethnic minority background. Ethnic minorities were defined as those having both parents born in a country other than Norway [32]. The parents' countries of birth were provided by Statistics Norway and were linked to the data file. Most of the ethnic minority youth (96%) came from non-Western countries. The largest ethnic minority groups were from the Indian subcontinent (42%), the Middle East (16%), and Eastern Europe (11%). Fifty-four percent of the minority group were born in a country other than Norway (first-generation immigrants).

### Measures

#### Weekly hours of physical activity in leisure time

Participants were asked how many hours per week they spent in physical activity "to an extent that makes you sweat and/or out of breath": 0 (0), 1–2 (1.5), 3–4 (3.5), 5–7 (6), 8–10 (9), or 11(11) hours or more per week. The numbers in the parentheses represent the coding when using the ranked ordinal variable as a continuous variable. In an unpublished validation study, physical activity was measured both objectively, using Actigraph accelerometers, and subjectively using the questionnaire. The question about "hours per week" was the one that best predicted physical activity measured by an accelerometer and was therefore chosen as our physical activity measure (Hein Stigum, Norwegian Institute of Public Health, personal communication). *Change *in physical activity was defined as the difference in hours of physical activity per week between baseline and follow-up. To study *stability *in physical activity from baseline to follow-up, we dichotomized hours per week spent doing physical activity as 0–2 hours versus 3 hours or more, and we measured the tendency of subjects to report the same activity level at the two times.

#### Socio-demographic factors

To obtain information on the education and income levels of fathers and mothers, the questionnaire was linked to information collected by Statistics Norway. Statistics Norway's register on income and education (2002) was used. In the analysis, education was categorized by the highest level of education: tertiary education, intermediate education, and compulsory education [33]. The father's and mother's income [34] was categorized as high (above the 75^th ^percentile), medium (25^th ^to 75^th ^percentile), or low (below the 25^th ^percentile).

The "perceived family economy" question had four options – "poor ", "moderate", "good", or "very good" – based on a question asking the participant to compare his or her family economy with other families in Norway. "Parents' marital status" was categorized as having parents who were married/cohabitant or "other" (i.e. divorced/separated, one or both deceased). "Socio-economic region of residence" in Oslo (neighbourhood socio-economic level) was a social index that took into account the distribution of unemployment, education, non-Western immigrants and single parents [35]. In our analyses, East and West regions were used.

#### Invitation group

Because the follow-up part of the study was conducted partly in school and partly by mail, a variable called "*Invitation group*", was created to dichotomize mail and school participation.

### Ethics

Both protocols were evaluated by the Regional Committee for Medical Research Ethics and were approved by the Norwegian Data Inspectorate. The baseline study and the part of the follow-up study carried out in the schools received approval from the school authorities in Oslo.

### Lost to follow-up

Seventy percent of the ethnic Norwegian students and 54% of the ethnic minority students participated in the follow-up study. In ethnic Norwegian participants, baseline physical activity did not differ between those who participated in the follow-up and those who were lost to follow-up. In the ethnic minority participants, physical activity level at baseline was higher in those who completed the follow-up than in those lost to follow-up. To test whether the physical activity level among the immigrants could be biased by the length of stay in Norway, we compared the physical activity level of those lost to follow-up and in those participating at both times in first- and second-generation immigrants. Weekly hours of physical activity by both boys and girls did not differ between first- and second-generation immigrants. To predict how attrition might bias the results, others have shown that subjects who participate after reminders are fairly similar to the non-responders [36]. In our analysis, the change in physical activity did not differ significantly between adolescents from ethnic minorities who participated after the first invitation and those who participated after reminders [mean difference in change in weekly hours of physical activity (SE), -0.28 (0.59), p = 0.59].

### Statistical analysis

The data were stratified by sex and partly by ethnicity (ethnic Norwegians and ethnic minorities). To compare physical activity levels at baseline (T1) and follow-up (T2), the change in physical activity from baseline to follow-up (T2-T1), and the associations between physical activity and socio-demographic factors at the two times, we classified hours per week as a continuous variable and analysed the data using analysis of variance (ANOVA). The patterns of association were compared between ethnic Norwegians and ethnic minorities by testing the interaction terms.

Cohen's kappa and percentage of agreement were used to study the stability in physical activity (0–2 hours versus 3 hours or more) from baseline to follow-up in the four subgroups.

Finally, we studied the association between socio-demographic factors and change in physical activity level (T2-T1) using a linear regression model. The estimated regression coefficients (*β*) and 95% confidence interval (95% CI) are presented in the text. The socio-demographic factors that showed associations with physical activity at T1 or T2 were entered collectively in the adjusted model. Only participants who provided information on all variables in the adjusted analysis were included in the crude comparisons. Weekly physical activity level did not differ significantly between participants excluded and those included in this analysis. The level of significance was set at p < 0.05. The data were analysed using SPSS version 14.

## Results

### Physical activity at 15 and 18 years

Boys were more physically active than girls at both 15 and 18 years, and this was independent of ethnic background (Table 1). We found ethnic differences in physical activity levels in girls but not boys. At both ages 15 and 18 years, ethnic Norwegian girls were more physically active than ethnic minority girls (Table 1).

**Table 1:** Physical activity level at age 15 and 18 years in ethnic Norwegian and ethnic minority adolescents.

	**Physical activity**								
		
	**Ethnic Norwegian**				**Ethnic minority**				
	***n***	**Mean**	95%CI*		***n***	**Mean**	95%CI*		***p *ethnicity**
**Boys**									
15 years (baseline)	*892*	5.35	5.13	5.58	*211*	5.00	4.55	5.46	*0.18*
18 years (follow-up)	*897*	4.61	4.38	4.84	*209*	4.37	3.92	4.83	*0.38*
Δ ** (follow-up-baseline)	*889*	-0.73	-0.96	-0.58	*209*	-0.61	-1.17	-0.06	*0.67*
**Girls**									
15 years (baseline)	*1058*	4.00	3.82	4.18	*283*	2.43	2.16	2.70	*< 0.001*
18 years (follow-up)	*1066*	3.58	3.41	3.77	*287*	2.01	1.74	2.27	*< 0.001*
Δ ** (follow-up-baseline)	*1044*	-0.38	-0.56	-0.20	*274*	-0.47	-0.77	-0.16	*0.66*

*95% confidence interval (CI).

** Δ Change from baseline to follow-up.

### Physical activity and socio-demographic factors at 15 and 18 years

Ethnic minority adolescents had parents with lower income and lower education level than ethnic Norwegians (p < 0.001). Compared with their Norwegian counterparts, more ethnic minority boys and girls lived in eastern regions of Oslo and had parents that were married/cohabiting (both sexes p < 0.001). Ethnic minority girls perceived poorer family economy than did ethnic Norwegian girls (p = 0.01). No difference was observed in boys (p = 0.38).

#### Boys

At age 15 years, ethnic Norwegian boys who perceived a poor family economy reported low physical activity level (Table 2a). At age 18 years, ethnic minority boys living in the eastern part of Oslo were more physically active than those living in the western part. The association between physical activity level and socio-demographic factors did not differ between ethnic Norwegian and ethnic minority boys at ages 15 or 18 years (i.e. no interaction).

**Table 2:** 

**2a: Physical activity level and socio-demographic factors in ethnic Norwegian and ethnic minority boys.**												
Boys (1112*)	**Physical activity at age 15 years**						**Physical activity at age 18 years**					
	**Ethnic Norwegian (900)**			**Ethnic minority (211)**			**Ethnic Norwegian (900)**			**Ethnic minority (211)**		
	
**Socio-demographic factors**	**M°**	**SD°**	**p**	**M°**	**SD°**	**p**	**M°**	**SD°**	**p**	**M°**	**SD°**	**p**

Income father ^#^			*0.12*			*0.69*			*0.91*			*0.17*
Low	3.98	3.30		4.80	3.54		4.03	3.34		5.37	3.34	
Medium	4.50	3.31		5.14	3.23		4.75	3.57		5.43	3.52	
High	6.04	3.88		4.46	3.33		4.65	3.38		5.32	3.36	
Education mother ^#^			*0.13*			*0.29*			*0.31*			*0.16*
Compulsory	4.43	3.64		5.34	3.51		3.92	3.69		4.50	3.31	
Intermediate	5.36	3.63		4.43	3.37		4.59	3.62		4.04	3.50	
Tertiary	5.44	3.25		5.28	3.21		4.69	3.37		5.47	3.26	
Perceived family economy			*0.01*			*0.54*			*0.22*			*0.74*
Poor	3.56	3.34		6.86	3.34		3.83	3.61		3.71	4.37	
Moderate	5.03	3.61		4.91	3.75		4.38	3.50		4.11	3.20	
Good	5.41	3.30		4.94	3.18		4.62	3.38		4.62	3.42	
Very good	5.96	3.54		5.05	3.42		5.10	3.83		4.21	3.44	
Parents marital status			*0.08*			*0.70*			*0.20*			*0.29*
Married or cohabiting	5.49	3.39		5.06	3.35		4.71	3.52		4.25	3.35	
Other ^§^	5.05	3.48		4.80	3.46		4.38	3.42		4.95	3.40	
Residence in Oslo			*0.15*			*0.79*			*0.01*			*0.93*
East	5.54	3.65		4.94	3.43		4.99	3.78		4.27	3.31	
West	5.20	3.24		5.13	2.77		4.38	3.24		4.34	3.73	

° Values are expressed as Mean (M) and Standard Deviation (SD). * Some participants have missing information on some of the background characteristics. # Information collected by Statistics Norway (2002). § Parents divorced or separated, or one or both parents dead.												

**2b: Physical activity level and socio-demographic factors in ethnic Norwegian and ethnic minority girls**												

Girls (1377*)	**Physical activity at age 15 years**						**Physical activity at age 18 years**					
	**Ethnic Norwegian (1081)**			**Ethnic minority (296)**			**Ethnic Norwegian (1081)**			**Ethnic minority (296)**		
	
**Socio-demographic factors**	**M°**	**SD°**	**p**	**M°**	**SD°**	**p**	**M°**	**SD°**	***p***	**M°**	**SD°**	**p**

Income father ^#^			*0.02*			*0.45*			*0.05*			*0.05*
Low	3.36	2.92		2.25	2.08		3.10	3.09		1.71	2.19	
Medium	4.00	3.04		2.40	2.52		3.55	3.02		2.00	2.24	
High	4.18	2.90		3.00	2.20		3.83	2.92		3.08	2.11	
Education mother ^#^			*0.74*			*0.32*			*0.04*			*0.12*
Compulsory	3.80	3.04		2.48	2.46		2.74	2.62		1.69	1.86	
Intermediate	4.07	2.96		2.29	2.20		3.55	3.05		2.35	2.66	
Tertiary	3.97	3.01		2.93	2.48		3.73	2.99		2.16	2.09	
Perceived family economy			*0.01*			*0.10*			*0.001*			*0.54*
Poor	3.30	2.80		2.21	3.25		2.25	2.58		1.07	0.73	
Moderate	3.56	2.99		2.07	1.91		3.13	2.90		1.94	2.33	
Good	4.11	2.92		2.48	2.34		3.73	2.99		2.17	2.23	
Very good	4.53	3.20		3.16	2.70		4.19	3.21		1.82	2.35	
Parents marital status			*0.11*			*0.12*			0.01**			0.14**
Married/cohabiting	4.10	2.97		2.33	2.24		3.77	3.00		1.88	2.31	
Other ^§^	3.79	3.00		2.85	2.51		3.24	2.98		2.36	1.93	
Residence in Oslo			*0.12*			*0.87*			*0.53*			*0.03*
East	4.15	3.09		2.42	2.36		3.65	3.13		1.89	2.17	
West	3.86	2.89		2.49	1.92		3.54	2.91		2.76	2.63	

° Values are expressed as Mean (M) and Standard Deviation (SD). * Some participants have missing information on some of the background characteristics. ** Interaction term = 0.02 (marital status, ethnicity, and physical activity). # Information collected by Statistics Norway (2002). § Parents divorced or separated, or one or both parents dead.												

#### Girls

In ethnic Norwegian girls, higher levels of physical activity at both ages 15 and 18 years were positively associated with father's income and perceived family economy. Physical activity at age 18 years was positively associated with mother's education and parents' marital status (Table 2b). Ethnic minority girls living in the western regions of Oslo were more physically active at age 18 years than were ethnic minority girls living in the eastern part (Table 2b). The associations between physical activity level and socio-demographic factors in the two ethnic groups were significantly different for only one factor: having married/cohabitating parents was positively associated with physical activity in Norwegian girls at age 18 years, but the opposite was the case in ethnic minority girls (Table 2b) (interaction term, p = 0.02).

The mother's income and father's education level was not significantly related to physical activity level in boys or girls.

### Change in and stability of physical activity from age 15 to 18 years

Physical activity level measured as weekly hours declined in all groups (Δ, change) from age 15 to 18 years (Table 1). The change in physical activity level from age 15 to 18 years did not differ between ethnic Norwegian and ethnic minority boys or girls (Table 1).

**Figure 2:**
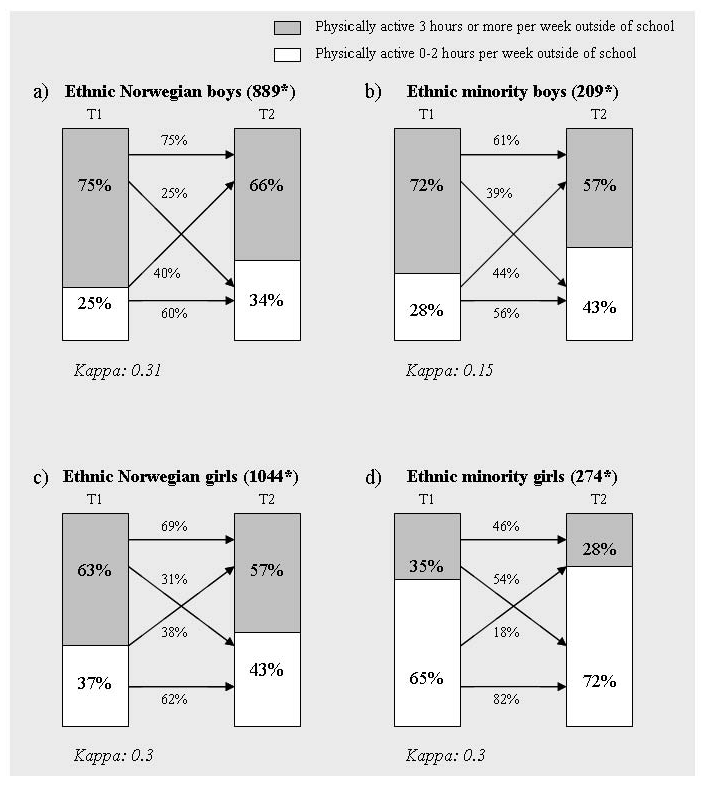
**Change in physical activity level from T1 to T2**. Percentage of participants who were physically active 0–2 hours per week or 3 or more hours per week at baseline (T1) and follow-up (T2), and percentage who maintained or changed activity level from T1 to T2 in ethic Norwegian (a and c) and ethnic minority (b and d) boys and girls. * _indicates the number of participants who answered the question about physical activity at both times._

More ethnic Norwegian girls and boys were physically active for three hours or more at both times (stability) than were ethnic minority girls and boys (Figure 2). Eighty-two percent of the ethnic minority girls had a persistently low physical activity level (0–2 hours per week) at both times. Fifty-four percent of ethnic minority girls who were physically active for more than three hours per week at age 15 years reduced their activity level at age 18 years (Figure 2). The kappa (κ) values for stability of physical activity level are presented in Figure 2 according to sex and ethnicity. The κ values varied between 0.15 and 0.31 for both sexes and ethnic groups. The lowest κ value was seen in ethnic minority boys.

### Socio-demographic factors and change in physical activity level over three years

We found similar associations in the crude and adjusted analyses for the association between socio-demographic variables and change in physical activity analysed by sex. The only significant associations found in the adjusted analyses were that physical activity level declined more in girls with mothers with compulsory education than in girls with mothers with tertiary education [*β*, -0.75; 95%CI, -1.38, -0.12)], and declined more in boys with fathers with low income than in boys with fathers with high income [*β*, -0.90; 95% CI, -1.66, -0.14).

The association between socio-demographic factors and change in physical activity level did not differ between ethnic Norwegians and ethnic minorities (i.e. no interaction).

## Discussion

### Physical activity and ethnicity

In our study, boys were more physically active than girls, as has been reported earlier [6,7,37]. We found ethnic differences in activity level, an observation that is consistent with previous findings from the UK [20] and USA [13,18], although other studies in the USA reported no differences [21] or higher physical activity level in some ethnic minority groups [16]. The difference between the USA and Europe may reflect differences in the immigrants' ethnicity, history, and time since immigration. Discrimination and racism are other factors that might cause differences in sport participation between ethnic minority and host adolescents [38,39]. Fear of being exposed to racism could be keeping ethnic minorities away from organized sport [40]. However, our results do not allow us to conclude whether racism influences the choice of physical activity type.

As observed in other studies [13,18], we found that ethnic differences in physical activity were more apparent in girls than in boys. Differences in physical activity may reflect the influence of factors such as the religion and culture in the country of origin. In our study, 96% of the ethnic minorities came from non-Western countries, the largest group being from the Indian subcontinent. The difference between boys and girls could relate to gender segregation in some religions (Islam), girls having more household responsibility, or stricter rules set by parents [41-43]. The ethnic minority girls' low activity level might also relate to the structure of organized sports in Norway, which includes fewer differences according to gender than in many of the ethnic minorities' countries of origin [44]. Another contributing factor could be the low physical activity level among ethnic minority women in Oslo [45]. Parents' physical activity patterns probably influence their children's physical activity through modelling, social influence, and social support [1].

### Physical activity and socio-demographic factors at age 15 and 18 years

Consistent with other studies, [24-26] we found few significant associations between socio-demographic factors and physical activity level. Such associations seem to be dependent on the SES measure used and the characteristic of the subgroup being studied.

One relatively consistent finding in our study was the lower physical activity level among ethnic Norwegians who perceived family economy as poorer than other Norwegian families. Those who perceived themselves as having low family income might be restricted in their physical activity choices and opportunities because of the cost involved.

The lack of association between physical activity and SES in ethnic minorities may indicate a different relationship between SES and health and disease, and a different influence on health behaviour in ethnic minorities than in ethnic Norwegians. A Norwegian study focusing on determinates of diabetes in different ethnic adult groups reported a negative association between the prevalence of diabetes and SES among ethnic Norwegians and Westerners but almost no association in ethnic minorities [45].

### Change in and stability of physical activity from age 15 to 18 years

We found a decline in mean hours per week of physical activity from age 15 to 18 years, which was similar in ethnic Norwegians and ethnic minorities. In the UK, Asian adolescents and black girls are less active than white girls at age 11–12 years, and this difference does not change over the next five years [20]. McMurray et al. [16] observed a similar decline in physical activity from age 8 to 16 years in African-American and Caucasian girls but a greater decline in Caucasian boys than in African-American boys. Another longitudinal study from the USA [13] found a substantial decline in physical activity level that was higher in black girls than in white girls.

The relatively low κ scores in our study suggest low stability of physical activity levels within groups. Anderssen et al. [12] used a similar physical activity questionnaire with youths in the western part of Norway and reported κ values, based on tertiles, of 0.26 for boys and 0.21 for girls over a three-year period (age 16–19 years). The only study that assessed physical activity stability in different ethnic groups (Caucasians and African-Americans) reported low κ values (0.03–0.22) [16]. The discrepancy in results might be because κ appears to be higher for shorter time periods, whereas our study and the study by Anderssen et al. [12] studied stability over a three-year period, and McMurray et al. [16] studied stability over seven years. We also dichotomized physical activity level at two times, whereas the other studies divided the physical activity variable into three groups.

McMurray et al. [16] also reported that more Caucasian than African-American youth remained in the low physical activity group during the follow-up. In our study, the highest percentage of persistently low physical activity was found among the ethnic minority girls. As suggested previously, the different findings might be explained by the different immigrant histories and cultures of origin of the ethnic minorities in the USA and in Norway. The discrepancy in results may also be caused by different sample sizes, age, and the definition of "low physical activity".

### Socio-demographic factors and change in physical activity

The only socio-demographic factors that were associated with change in physical activity level were mother's education in girls, and father's income in boys. The few associations observed might reflect the influence of more important circumstances experienced by this group of youth. These adolescents had experienced changes in schools, increasing homework level, and the biological, social, and psychological changes that accompany puberty. These factors, alone or in combination, may affect physical activity significantly and might "overrule" the importance of socio-demographic factors.

### Methodological issues

Measuring physical activity level by questionnaire is associated with difficulties [46]. The measure used to capture physical activity level in this study is general: "activities outside of school that make you feel sweaty and out of breath". Such a crude overall measure does not capture all the physical activities that promote health. However, simple, self-reported questions on overall physical activity have been used in several studies and correlated significantly with other activity measures [47,48], and with other indicators of physical activity such as maximal oxygen uptake [49] and physical fitness [50]. It seems reasonable to assume that the question captures the level of physical activity, although we do not know how accurate the adolescents were in reporting hours per week. However, we compared associations, change in, and stability of physical activity between groups and believe that the measure is reasonably reliable and valid for this purpose. Generally, the more unreliable the measure, the greater the chance of not finding differences or associations that exist. Therefore, we probably underestimated rather than overestimated any associations and difference between the groups.

Performing a large number of tests, as we did when studying the relationship between physical activity level and socio-demographic factors (aims 2 and 4), increases the risk of type 1 error. Hence, we emphasize the pattern of our findings and interpret the single significant associations with caution.

We also note the variety of ethnic minorities represented by our sample. Even though about 96% of the ethnic minorities in our study population were from non-Western countries, they were not a homogenous group. Studying all ethnic minorities together might conceal differences in physical activity levels between different ethnic groups.

The attrition is also a concern. Of those participating in the baseline study, 70% of the ethnic Norwegians and 54% of the ethnic minorities participated in the follow-up. Ethnic minorities participating at follow-up reported more physical activity at baseline than did the ethnic minorities lost to follow-up; there was no corresponding difference at baseline in the ethnic Norwegians. However, in the ethnic minorities, the physical activity level did not differ between those who completed the questionnaire after reminders compared with those who participated after the primary invitation, indicating that the selection probably did not influence the results substantially [36].

## Conclusion

This study confirms that boys are more physically active than girls, and the ethnic differences in physical activity are more pronounced in girls than boys. Further, the physical activity level declines during the late teenage years in all groups. Socio-demographic factors are only weakly related to physical activity level at age 15 and 18 years, and the change in physical activity between these ages. Ethnic minority girls were the least physically active group, and a considerable proportion reported a stable low activity level. Religious, cultural, social, and environmental factors may contribute to the differences and the decline in physical activity level. Society-wide approaches promoting physical activity that focus on families and local society, and that consider the special needs of subgroups such as immigrant girls, are required to reverse these patterns.

## Competing interests

The authors declare that they have no competing interests.

## Authors' contributions

ÅS was active in the planning of the follow-up and coordinated the practical part of the study, conceptualized and designed the study, analysed and interpreted the data, and drafted the manuscript. EK conceptualized and designed the study, discussed the analysis and interpretation of the data, and drafted the manuscript. SAA was involved in the conceptualization and design of the article, discussed the analysis and interpretation of the data, and reviewed the manuscript critically. MT was involved in the conceptualization and design of the article, contributed to the statistical analyses and interpretation of the data, and reviewed the article critically. AJS was project manager of the baseline study, participated in panning of the follow-up study, was involved in the conceptualization and design of the article, discussed the analysis and interpretation of the data, and reviewed the manuscript critically.

## Pre-publication history

The pre-publication history for this paper can be accessed here:


